# Case report: anti-fibrillarin autoantibodies induced by viral molecular mimicry in a paediatric patient

**DOI:** 10.1186/s13052-025-02029-0

**Published:** 2025-07-03

**Authors:** Chiara Autilio, Raffaele Pecoraro, Vito Pafundi, Sergio Manieri, Vincenzo Tipo, Luigi Martemucci, Teresa Carbone

**Affiliations:** 1 Regional San Carlo Hospital, Clinical Pathology and Microbiology Unit, Potenza, Italy; 2 Department of Biochemistry and Molecular Biology, Complutense University, Madrid, Spain; 3 Pediatric Emergency Department, Santobono-Pausilipon Children’s Hospital, Naples, Italy; 4 Pediatric Department, Regional San Carlo Hospital, Potenza, Italy; 5 Immunopathology Laboratory, Regional San Carlo Hospital, Potenza, Italy

**Keywords:** Fibrillarin, Autoantibodies, Molecular mimicry, Infection, Case report

## Abstract

**Background:**

Anti-fibrillarin autoantibodies (AFA) as serological hallmarks of systemic sclerosis, mainly react with epitopes arranged in the NH2-(aa-1–80) and COOH-terminal-(aa-276–321)-domains of fibrillarin. Interestingly, the fibrillarin NH2-hexapeptide sequence is shared with an Epstein-Barr-virus (EBV)-encoded nuclear antigen.

**Case presentation:**

We herein report a case of a 14-year-old girl presenting with a history of vomiting, sore throat, arthralgias and fever. Laboratory tests revealed leukocytosis, an increased level of CRP, transaminases and total/direct bilirubin. On further investigation, a positivity of ANA testing showing a clumpy nucleolar indirect immunofluorescence (AC-9) pattern on HEp-2000 substrate, due to anti-fibrillarin antibodies, was found. Concomitantly, high concentrations of EBV-VCA-IgM and a slight increase of EBV-VCA-IgG were detected, helping establish a diagnosis of ongoing EBV infection. After a follow-up of six months, all autoimmunity tests were repeated, and together with infection resolution, the negativity of ANA was determined, confirming the transient nature of the autoimmune phenomenon.

**Conclusions:**

Our findings confirm how molecular mimicry plays an important role in the viral-induced autoimmunity. Given the significant homology between fibrillarin and EBV protein sequences, caution in interpreting AFA positivity is suggested, especially in pediatric patients without clinical evidences of an autoimmune condition, and a simultaneous screening for EBV infections is recommended.

## Background

Molecular mimicry involving immunological cross-reactivities between self-antigens and microbial determinants is one of the leading processes which, in susceptible individuals, may induce autoimmunity [1]. The mechanism is triggered by the recognition of self-sequence of a known antigen by autoantibodies against viral or microbial (linear or conserved) epitopes, given their antigenic analogy. In a case of cross-reactivity, tissue-specific or systemic immune response eliciting cell and tissue destruction or transient autoimmune phenomena could occur. Studies showed homologies between human cytomegalovirus protein UL70 and Topoisomerase I, one of the main autoantigens in systemic sclerosis (SSc), supporting the hypothesis of the viral infection as a possible triggering factor of the disease, although no evidence has been provided [2]. Recent bioinformatic analyses revealed a significant homology between immunodominant peptides on topoisomerase I, fibrillarin, Centromere protein A (CENP-A) and proteins from viruses of the Mimiviridae and Phycodnaviridae families, suggesting a potential environmental link in SSc pathogenesis. Viral peptides could activate T-helper cells, which, in turn, stimulate B cells with receptors specifically recognizing these nuclear antigens leading to the development of anti-topoisomerase, anti-centromere or anti-fibrillarin antibodies (AFA) [3].

**Fig. 1:**
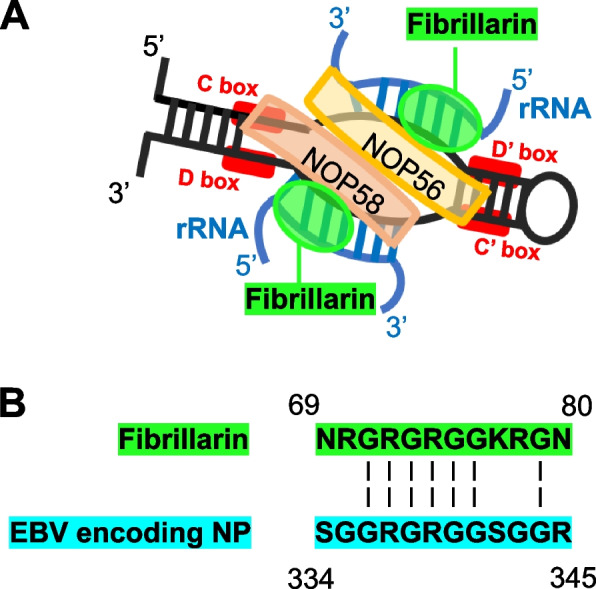
**A** Schematic model of U3 snoRNP structure containing fibrillarin. **B** Hydrophilic fibrillarin peptide motifs (potential antigenic sites) exhibiting sequence similarity with a nuclear protein encoded by EBV genome (adapted from [10]). rRNA: ribosomal RNA; EBV: Epstein-Barr-Virus; NOP56: Nuclear Protein 56; NOP58: Nuclear Protein 58

Fibrillarin is a highly conserved 34-kDa basic protein which is localized in nucleoli [4]. It is the main antigenic determinant of human U3 RNP (Fig. 1A) and a molecular marker of transcriptionally active RNA polymerase I [5]. The protein is characterized by three different domains: 1) NH2-terminal domain of 80 amino acids rich in glycine and dimethylarginine, 2) central domain of 90 amino acids carrying the RNA binding consensus sequence and 3) COOH-terminal alfa helical domain of 151 amino acids [4, 6].

Anti-fibrillarin autoantibodies as serological hallmarks of systemic sclerosis showed a frequency ranging from 7–48% across different ethnic groups. AFA were mainly mutually exclusive of the major SSc associated antibodies [7]. Systemic sclerosis, a rare disease in childhood, is characterized by skin fibrosis, internal organ involvement and vasculopathy. Clinically, SSc patients with AFA have been reported to have higher frequency of diffuse cutaneous involvement, pulmonary arterial hypertension, musculoskeletal and cardiac involvement and lower frequencies of arthritis [8]. Juvenile systemic sclerosis (JSSc) represents less than 10% of all scleroderma patients. The onset of JSSc is usually insidious. Isolated Raynaud phenomenon is often the presenting symptom along with positive ANA and nailfold capillary changes. As compared with adults, children with JSSc have overall better outcomes, related to a lower frequency of major visceral organ involvement and lower mortality. As opposed to adult patients with systemic scleroderma, specific autoantibodies are not common in the paediatric form of the disease. Only one JSSc AFA positive patient was found in a small cohort of children who met classification criteria for JSSc [9].

Specifically, AFA seem to react primarily with epitopes present in the NH2-(aa-1–80) and COOH-terminal-(aa-276–321)-domains of fibrillarin. Interestingly, as described in literature and shown in Fig. 1B, the fibrillarin NH2-hexapeptide sequence is shared with an Epstein-Barr-virus (EBV)-encoded nuclear antigen [10]. However, no incidental findings of EBV molecular mimicry inducing AFA positivity have been described to date.

Here, we report the first case, to our knowledge, of a transient AFA positivity induced by EBV mimicry in a paediatric patient.

## Case presentation

A 14-years-old girl of Caucasian origins was admitted to the Emergency Department (ED) of the Regional San Carlo Hospital, Potenza (Italy), with a history of vomiting, sore throat and fever started six days before. She had only taken ibuprofen as needed. At physical examination exudative pharyngitis and bilateral cervical lymphadenopathy were found. In the patient’s medical history, recurrent episodes of arthralgias, oral ulcers, anorexia and fatigue were described.

Laboratory analyses revealed normal hemoglobin and platelets, leukocytosis with a high lymphocytes and monocytes count, an increased level of C-Reactive Protein (CRP), transaminases, lactate dehydrogenase (LDH) and total/direct bilirubin with cholestasis (Table 1). The abdominal ultrasonography revealed hepato-splenomegaly, enlargement of the lymph nodes of the hepatic hilum and periportal edema. Hepatotropic viruses and, for the past medical history, serologic autoantibodies for celiac disease, anti-smooth muscle antibodies (ASMA), anti-Liver-Kidney Microsomial (LKM1) and anti-nuclear antibody (ANA) screening were required to ascertain the cause of the cholestatic hepatitis.

**Table 1:** Laboratory tests at patient’s ED admission and after six months of follow-up

	**ED admission**	**Follow-up**	**Reference range**	**Analyzer**
Blood count				
Red blood cell count	4.2	5.2	3.9—5.2 × 10^6/µL	Sysmex XN-3000 [Sysmex]
Hemoglobin	12.4	14.4	12.0—15.6 g/dL	
White blood cell count	12.2	6.6	4.7—10.7 × 10^3/µL	
Neutrophils	17.8	65.5	49.0—80.0%	
Lymphocytes	63.1	29.6	12.0—37.0%	
Clinical chemistry				
CRP	30.7	0.3	0.0—5.0 mg/L	DxC-700-AU [Beckman coulter]
GOT	233	15	5—50 U/L	
GPT	250	21	5—50 U/L	
LDH	580	222	0—248 U/L	
GGT	107	19	5—38 U/L	
ALP	331	95	34—120 U/L	
Total/Direct bilirubin	4.5 / 2.70	0.9 / 0.20	0.3–1.2 / 0.01–0.20 mg/dL	
EBV screening				
EBV-VCA-IgM	> 160	16.4	< 20.0 UA/mL	LIAISON® XL [Diasorin]
EBV-VCA-IgG	29.6	49.3	< 20.0 UA/mL	
EBNA-IgG	< 3.0	25	< 5.0 UA/mL	
CTD SCREEN*				
CTD SCREEN	4.3	0.8	< 1.0 Ratio	Phadia 250 [Thermofisher]

*CRP* C-Reactive Protein, *GOT* Glutamate Oxaloacetate Transaminase, *GPT* Glutamate Pyruvate Transaminase, *LDH* Lactate Dehydrogenase, *GGT* Gamma-Glutamyl Transferase, *ALP* Alkaline Phosphatase, *EBV-VCA-IgM* anti-Epstein-Barr virus capsid antigen Immunoglobulin M, *EBV-VCA-IgG* Anti-Epstein-Barr virus capsid antigen Immunoglobulin G, *EBNA-IgG* Anti-Epstein-Barr Nuclear Antigen Immunoglobulin G, *CTD* Connective Tissue Disease

^*^ ELiA CTD screen contains a mixture of human recombinant U1RNP (RNP70, A,C), SS-A/Ro (60 kDa, 52 kDa), SS-B/La, Centromere B, Scl-70, Jo-1, fibrillarin, RNA Pol III, Rib-P, PM-Scl, PCNA, Mi-2, Sm and native purified DNA

**Fig. 2:**
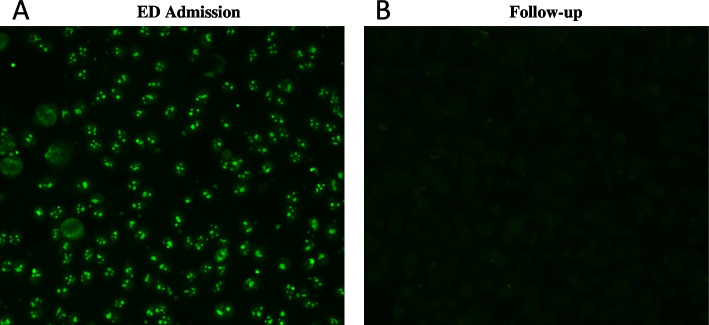
**A** ANA positivity showing clumpy nucleolar indirect immunofluorescence pattern (AC-9) at patient’s ED admission. **B** ANA negativity after a follow-up of six months. ED: Emergency Department

All autoantibody determinations were conducted in the Immunopathology Laboratory of the Regional San Carlo Hospital, Potenza (Italy). ANA test was performed at 1:160 screening dilution on automated analyzer (Gemini Combo, Stratec Biomedical) using commercial ANA HEp-2000 Indirect Immunofluorescent assays (HEp-2000 Fluorescent ANA-Ro Test System, Immuno Concepts N.A., Sacramento, CA, USA) and anti-human IgG specific fluorescein-labelled conjugate according to the manufacturer’s instructions. Interestingly, the positivity of ANA testing showing a clumpy nucleolar indirect immunofluorescence (AC-9) staining pattern at 1:320 end-point titer, was observed (Fig. 2A), compatible with the presence of antibodies against U3-snoRNP/fibrillarin antigen [11, 12]. The patient was also tested for ELiA Connective Tissue Disease (CTD) assay (Thermo Fisher Scientific, Freiburg, Germany) revealing a positive result above the cut-off value (Table 1). To confirm the presence of SSc-related autoantibodies, the Immunoblotting Scleroderma Profile (Alifax, Polverara, Italy) was performed, confirming an isolated positivity of anti-fibrillarin antibodies, corresponding to the morphological features of the observed staining pattern. Anti-ASMA and anti-LKM antibodies, performed at 1:40 screening dilution on liver-kidney-stomach rat tissues, resulted negative.

Concomitantly, high concentrations of anti-Epstein-Barr virus capsid antigen Immunoglobulin M (EBV-VCA-IgM) and a slight increase of anti-Epstein-Barr virus capsid antigen Immunoglobulin G (EBV-VCA-IgG) along with a negative anti-Epstein-Barr Nuclear Antigen Immunoglobulin G (EBNA-IgG) were detected, helping establish a diagnosis of ongoing EBV infection (Table 1).

Due to the unusual occurrence of anti-fibrillarin autoantibodies in pediatric patients and the concomitantly EBV infection, a comment in the report was included as follows: ‘*The positivity of anti-fibrillarin autoantibodies represents a rare finding in paediatric patients, thus requiring a rheumatological evaluation. However, given the ongoing EBV infection and the homology of viral sequences and fibrillarin autoantigens, a case of EBV molecular mimicry should be taken into account*’.

The rheumatological evaluation excluded an ongoing autoimmune condition given the unmet classification criteria of SSC: skin thickening of the fingers of both hands extending proximal to the metacarpophalangeal joints, sclerodactyly of the fingers, digital tip ulcers, telangiectasia, pulmonary arterial hypertension and/or interstitial lung disease, Raynaud’s phenomenon and SSc-related autoantibodies (anticentromere, anti–topoisomerase I [anti–Scl-70], anti–RNA polymerase III). A family history of autoimmune disease was also ruled out [13, 14]. During the hospitalization, the patient was treated with supportive care (antipyretics, hydration, and rest) and oral prednisone therapy administered for twelve days (the first three days at a dosage of 1 mg/kg/die, followed by a progressive tapering over the next nine days until discontinuation) with a prompt resolution of symptoms. After the discharge from the hospital, a posthospitalization follow-up was prescribed.

After a follow-up of six months, the absence of suggestive clinical signs of SSc was confirmed. All autoimmunity and infective serological tests were repeated, and together with infection resolution, the negativity of ANA (Fig. 2B), CTD screen and immunoblotting was determined (Table 1), confirming the transient nature of the autoimmune phenomenon.

## Discussion and conclusions

Here, we present a case of paediatric patient with a transient positivity of autoantibodies against fibrillarin, induced by a molecular mimicry mechanism triggered by a concomitant EBV infection.

Autoimmune responses to autoantigens can be induced by antigenic mimicry between autoantigen peptides and peptides from foreign antigens, such as viruses, bacteria or fungi. According to the theory of molecular mimicry, some antigens in the pathogen structure may be homologue to those of the host [1]. In these cases, the immune cells initially activated against the pathogen during infection, by a mechanism of cross-reactivity, recognize the self-components as potential targets and generate a systemic or organ-specific autoimmune response. The ongoing infection can also lead to autoimmunity through the presentation of cryptic antigens. Under normal conditions, these antigens are not exposed to the immune system, but during the inflammatory reaction, the action of proteases was increased and may lead to a presentation of self-antigens that induces an autoimmune response [4]. In this context, a strong association between rotavirus infections and celiac disease, autoimmune diabetes and autoimmune uveitis was described [15–19]. Autoimmune-related side effects of viral vaccines, particularly those based on adjuvants, have also been reported [20, 21]. A previous study on human autoimmune polyendocrinopathy-candidiasis-ectodermal dystrophy (APECED) showed a pathogenic connection between autoreactive T cells, fungal infection and carcinogenesis, also confirmed in a mouse model [22].

However, it is still under discussion if the structural homology between viral and self-antigen is linked to an early and transient or long-lasting autoimmune phenomenon.

Our clinical case also highlighted how the interdisciplinary approach based on the collaboration between clinicians and autoimmunologists, may play a crucial role in the correct interpretation of clinical and laboratory findings. Indeed, the interpretative competence of the autoimmunologist related to the recognition of rare or uncommon ANA positivity, as well as the managing of unexpected results, assumed a decisive weight in guaranteeing efficacy throughout the diagnostic process [23, 24].

Further studies involving a larger cohort of patients are needed to investigate the relationship between AFA development, the EBV and related viral agent infections, the genetic background and the gender of patients. Although a transient nature of AFA emerged, a longer follow-up has been planned for our pediatric patient, in order to evaluate the clinical conditions as well as the autoimmune serological status over time.

In conclusion, given the significant homology between fibrillarin and viral protein sequences from EBV, to avoid unnecessary further tests and potentially harmful therapy, caution in interpreting AFA positivity in pediatric patients and a simultaneous screening for EBV infections are recommended.

## Data Availability

All data generated or analysed during the current case are included in this published article.

## Abbreviations

AFA: Anti-fibrillarin autoantibodies
EBV: Epstein-Barr-virus
SSc: Systemic sclerosis
JSSc: Juvenile systemic sclerosis
ED: Emergency Department
RR: Reference range
CRP: C-Reactive Protein
GOT: Glutamate Oxaloacetate Transaminase
GPT: Glutamate Pyruvate Transaminase
LDH: Lactate Dehydrogenase
GGT: Gamma-Glutamyl Transferase
ALP: Alkaline Phosphatase
ASMA: Anti-smooth muscle antibodies
LKM1: Anti-Liver-Kidney Microsomial 1
ANA: Anti-nuclear antibody
CTD: Connective Tissue Disease
EBV-VCA-IgM: Anti-Epstein-Barr virus capsid antigen Immunoglobulin M
EBV-VCA-IgG: Anti-Epstein-Barr virus capsid antigen Immunoglobulin G
